# System for automatic gait analysis based on a single RGB-D camera

**DOI:** 10.1371/journal.pone.0201728

**Published:** 2018-08-03

**Authors:** Ana Patrícia Rocha, Hugo Miguel Pereira Choupina, Maria do Carmo Vilas-Boas, José Maria Fernandes, João Paulo Silva Cunha

**Affiliations:** 1 Institute of Electronics and Informatics Engineering of Aveiro (IEETA), and Department of Electronics, Telecommunications and Informatics, University of Aveiro, Aveiro, Portugal; 2 Institute for Systems Engineering and Computers – Technology and Science (INESC TEC), and Faculty of Engineering (FEUP), University of Porto, Porto, Portugal; Berner Fachhochschule, SWITZERLAND

## Abstract

Human gait analysis provides valuable information regarding the way of walking of a given subject. Low-cost RGB-D cameras, such as the Microsoft Kinect, are able to estimate the 3-D position of several body joints without requiring the use of markers. This 3-D information can be used to perform objective gait analysis in an affordable, portable, and non-intrusive way. In this contribution, we present a system for fully automatic gait analysis using a single RGB-D camera, namely the second version of the Kinect. Our system does not require any manual intervention (except for starting/stopping the data acquisition), since it firstly recognizes whether the subject is walking or not, and identifies the different gait cycles only when walking is detected. For each gait cycle, it then computes several gait parameters, which can provide useful information in various contexts, such as sports, healthcare, and biometric identification. The activity recognition is performed by a predictive model that distinguishes between three activities (walking, standing and marching), and between two postures of the subject (facing the sensor, and facing away from it). The model was built using a multilayer perceptron algorithm and several measures extracted from 3-D joint data, achieving an overall accuracy and F_1_ score of 98%. For gait cycle detection, we implemented an algorithm that estimates the instants corresponding to left and right heel strikes, relying on the distance between ankles, and the velocity of left and right ankles. The algorithm achieved errors for heel strike instant and stride duration estimation of 15 ± 25 ms and 1 ± 29 ms (walking towards the sensor), and 12 ± 23 ms and 2 ± 24 ms (walking away from the sensor). Our gait cycle detection solution can be used with any other RGB-D camera that provides the 3-D position of the main body joints.

## Introduction

Human gait analysis is the systematic study of human walking [1]. Quantitative gait information can be very useful in sports, biometric identification [2–6], and healthcare (e.g., for supporting the assessment of patients with gait impairments [7–9], both in clinical and at-home settings).

With the advent of sensors and their widespread use in our daily lives, many studies on gait recognition and/or analysis have been relying on sensors, such as wearable sensors (e.g., accelerometers, gyroscopes) [2, 3, 10–15] and vision-based sensors (e.g., RGB, RGB-D, and infrared cameras) [3–6, 16–22]. Regarding the detection of gait cycles, several solutions have been proposed in the area of computer vision [17, 18, 21, 23–31].

Some of these solutions use marker-based motion capture systems (e.g., Vicon, Qualysis) [23–27], which require the use of several infrared cameras, as well as retroreflective markers placed at the different body parts. As an alternative to these complex and expensive motion capture systems, the use of a single RGB camera and paper markers has been proposed [28]. However, this method has the disadvantage of allowing only 2-D joint tracking.

Recently, low-cost RGB-D cameras, such as the Microsoft Kinect [32], have emerged. These cameras provide not only color images, but also infrared and/or depth information. Based on the depth information, the Kinect is able to perform 3-D tracking of several body joints in a markerless way. Thus, it is not only more affordable and portable, but also less intrusive, when compared with motion capture systems that use several cameras and/or markers. For these reasons, RGB-D cameras have often been used in the development of solutions for gait cycle detection and/or gait analysis [17, 18, 21, 29–31].

Studies on gait usually assume that the subject carried out only the walking activity when the data were acquired. However, in a real situation, even if the subject is instructed to perform a simple and well-defined gait task, they may also carry out other activities (e.g., stop and stand still for a given amount of time). Therefore, automatic gait analysis should include the detection of the walking activity.

Gait analysis consists of the computation of parameters for each gait cycle [1], such as gait cycle (or stride) duration and length, gait speed, and cadence. A gait cycle corresponds to the time interval between two successive occurrences of a walking event, typically the instant at which one foot contacts the ground, which corresponds to a heel strike [1]. When using this reference, gait parameter computation depends on identifying the instants corresponding to heel strikes of the same foot. Therefore, automatic heel strike detection is essential for performing gait analysis without any external/manual intervention.

In our previous contributions, we verified that gait parameters extracted from 3-D joint data provided by the Kinect can possibly be used to distinguish between Parkinson’s disease patients and healthy subjects. However, in these studies the gait cycles were identified manually by visualizing color images acquired at the same time as the 3-D body joint data.

Therefore, we propose a system for fully automatic gait analysis using a single RGB-D camera, namely the second version of the Kinect (Kinect v2). Our system does not require any manual or external intervention (besides starting and stopping the data acquisition), since it automatically detects the walking activity. When walking is detected, the different gait cycles performed by the subject are then automatically identified, and several gait parameters are extracted for each gait cycle (e.g., gait cycle or stride duration and length, and gait speed).

Regarding walking recognition, our system is also able to detect whether the subject is walking towards or away from the sensor. This ability is important, since the Kinect itself assumes that the subject is always facing the sensor. This means that when the subject is facing away from it, the left joints are incorrectly tracked as the right joints, and vice-versa. Therefore, when we wish to obtain left and right gait parameters separately from data including both walking towards and away from the sensor, it is necessary to detect the “walking away” situation and correct the positions of left and right joints. The ability to detect between the two types of walking is also useful if we wish to use only the data corresponding to walking towards the sensor, for example.

The solution for gait cycle detection (including activity recognition) has also the advantage that it can be used online, allowing the verification of the number of gait cycles detected up to a given instant during a data acquisition. This is very useful when we wish to carry out gait analysis based on a minimum number of gait cycles, since it helps to save time by avoiding additional acquisitions. When using an offline solution, new acquisitions would be necessary if the desired number of gait cycles is not obtained. The proposed solution was developed and evaluated using a dataset corresponding to twenty healthy subjects. The dataset includes 3-D body joint data acquired concurrently using a RGB-D camera, and a gold standard motion capture system (Qualysis system with twelve infrared cameras).

The walking activity detection relies on 38 different measures computed from the 3-D joint data, and a predictive model that recognizes three different activities: walking, standing, and marching. The model also recognizes if the subject is facing the sensor or not. To find the best predictive model, different machine learning algorithms were explored: k-nearest neighbors, classification tree, random forest, support vector machines, multilayer perceptron, and multilayer perceptron ensemble.

The detection of gait cycles is carried out by estimating the instants corresponding to left and right heel strikes, relying on three measures extracted from 3-D data: distance between ankles, and velocity of both left and right ankles. The error for estimating the heel strike instants, as well as the spatiotemporal gait parameters (stride and step duration and length, and gait speed), was evaluated by comparing the estimated values with the corresponding actual values obtained using Qualysis data. This evaluation was performed for walking towards and away from the sensor, to investigate whether the algorithm performs differently for these two situations. To the best of our knowledge, this study has never been performed before.

## Related work

In the past few years, RGB-D cameras have been used in several studies to carry out human activity recognition [33–37]. However, most of these studies focus on the recognition of gaming actions (e.g., wave, punch, kick, clap, jogging) [33, 37], or very specific daily life activities (e.g., talking on the phone, drinking water, working on computer, cooking) [34–36], where standing and/or walking are often considered as random activities.

In the context of gait analysis, different methods for gait cycle detection using the Kinect were proposed [17, 18, 21, 29–31, 38]. With the aim of gait rehabilitation in Parkinson’s disease, Cancela et al. implemented a finite-state machine that detects different gait cycles phases [29], based on the left and right foot position provided by the first version of the Kinect (Kinect v1). For a dataset corresponding to seventeen healthy subjects, the detection error (i.e., percentage of steps not detected) ranged between 6% and 75%, for different walking paths and rhythms, as well as different sensor heights.

A state machine was also used by Gabel et al. for identifying gait cycles [18]. However, they used the output of a predictive model, which detects whether the foot is in contact with the ground or not, as the input of the state machine. The model was built using the Multiple Additive Regression Trees algorithm, and features computed over data acquired from twenty-three healthy subjects. The proposed solution achieved an estimation error of 8 ± 62 ms and 2 ± 46 ms for the duration of the left and right strides, respectively (actual duration obtained from pressure sensor data). The mean absolute error was of 45 and 32 ms, respectively.

With the aim of measuring stride-to-stride gait variability for fall risk assessment, Stone et al. proposed a solution for computing stride parameters using the Kinect v1 [17]. This solution includes the detection of left and right footfalls, which corresponds to finding the minima and maxima of a correlation coefficient. The latter was computed over normalized ground plane projections of 3D point cloud representing the subject’s silhouette. For data collected from three subjects, the proposed solution was able to compute the stride time with an error of 7 ± 62 ms (ground truth obtained from Vicon data).

Clark et al. proposed the identification of different strides by taking into account the toe-off instead of ground contact events [21]. The toe-off instants are estimated by finding the local minima of foot velocity that immediately precedes the instants when the foot velocity first exceeds a given threshold. For a dataset corresponding to twenty-one subjects, the stride time was estimated with mean error of −200 ms (ground truth obtained from Vicon data).

Instead of relying on the position of the feet, Auvinet et al. proposed the detection of heel strikes by finding the maxima of the distance between knees, along the longitudinal walking axis [30]. For the Kinect v1 sensor, the z-axis coordinate of the knees’ position is obtained from depth data by using k-means clustering. An estimation error of 17 ± 24 ms was achieved for heel strike instants, when considering data acquired from eleven healthy subjects, while they carried out treadmill walking (actual heel strike instants obtained from Vicon data). The estimation error for stride duration was of 0 ± 12 ms.

Another method for heel strike detection during treadmill walking was proposed by Xu et al. [31]. In this method, heel strike instants are estimated by finding the local maxima of the anterior-posterior distance between the hip center and ankle joints. For data collected from twenty healthy subjects, the method was able to estimate the stride duration with an error between 0 ± 19 ms and 2 ± 33 ms, depending on the walking speed of the subject.

Recently, Amini et al. used the Kinect v2 for detecting foot-off and foot contact events [38]. The foot-off events were detected by finding the instants for which the knee angle decreases to less than a given threshold. The foot contact events were detected by finding the instants for which the knee angle exceeds the same threshold value and the time interval between the instant and the last foot-off event is over 200 ms. An accuracy of 87% was achieved on average for different setups of the sensor, when considering data acquired from eleven healthy subjects.

## Materials and methods

### Subjects

An experimental study was conducted at LABIOMEP (Porto Biomechanics Laboratory) with the participation of twenty healthy subjects: ten male and ten female. The associated demographics are presented in Table 1. The experiment was approved by the Ethics Committee of Santo António hospital (Porto, Portugal), and all subjects signed an informed consent form.

**Table 1:** Characterization of the subjects that participated in the experiment.

	Mean ± SD	Minimum	Maximum
**Age (years)**	31 ± 8	23	52
**Height (m)**	1.71 ± 0.11	1.50	1.94
**Weight (kg)**	67.9 ± 15.3	48.0	105.0
**Body mass index (kg/m**^**2**^**)**	23.0 ± 3.3	16.7	31.0

SD stands for standard deviation.

The participants were recruited during the first half of June 2016. We approached a total of 25 subjects, including students, teachers and staff from the University of Porto (Portugal). Five out of the 25 subjects were not able to participate in the study due to unavailability on the dates scheduled for the data acquisitions. The only exclusion criterion for subject selection was the existence of any disease or injury that affected their gait (no limits were imposed regarding the age, height and weight).

### Experimental setup

**Fig 1:**
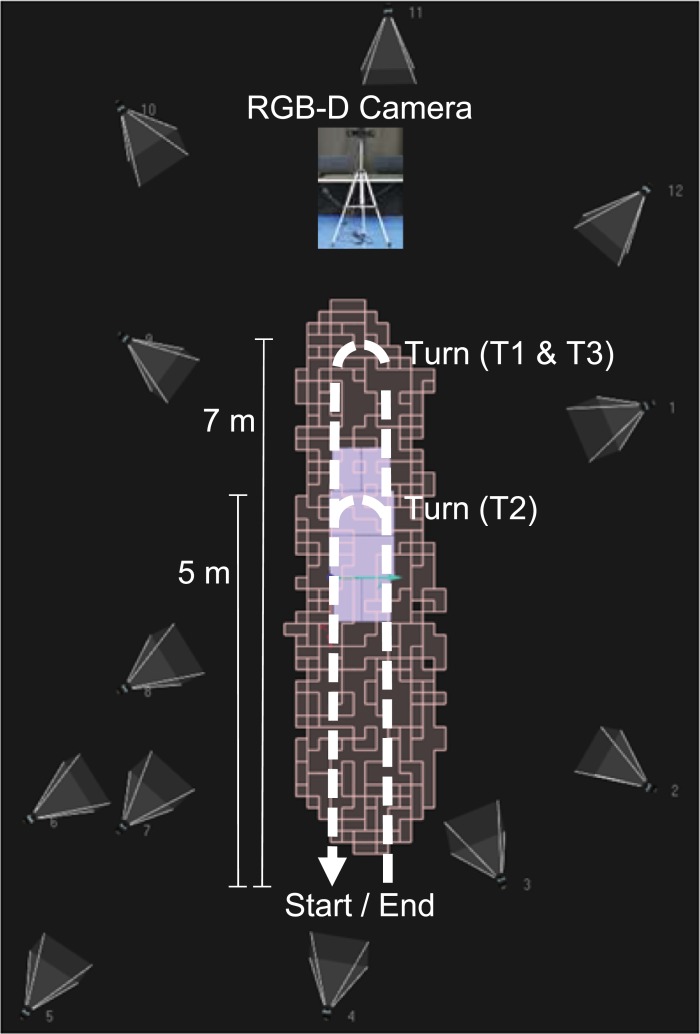
Experimental setup including a RGB-D camera (Kinect v2) mounted on a tripod, and a Qualysis system with twelve infrared cameras. The calibrated volume for Qualysis is illustrated by the salmon-coloured blocks. The walking path carried out by the subjects, for each task included in the protocol (T1, T2 and T3), is represented by the dashed arrowed lines. The relevant distances are also indicated. The figure was adapted from the Qualysis setup image provided by LABIOMEP (Porto Biomechanics Laboratory).

**Fig 2:**
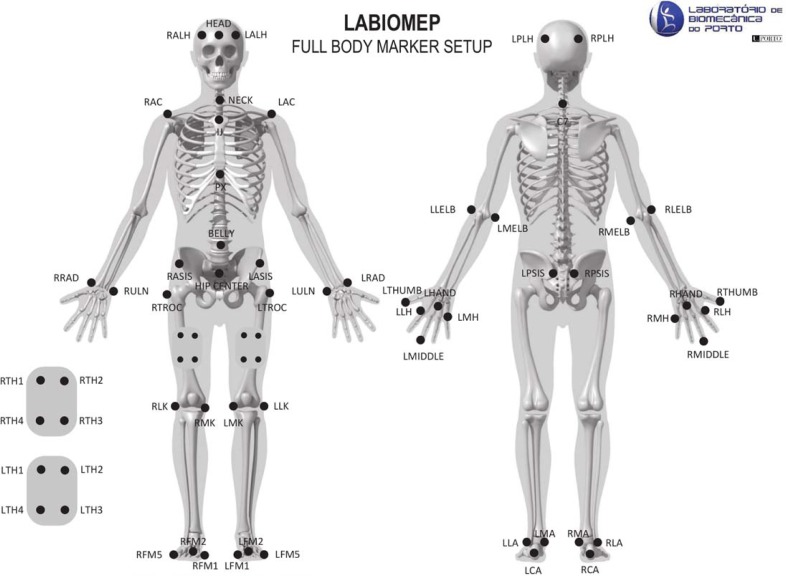
Retro-reflective markers placed at the subject's body. This figure was provided by LABIOMEP (Porto Biomechanics Laboratory).

The experimental setup is depicted in Fig 1. It included two different motion capture systems: a single RGB-D camera (Kinect v2); and a gold standard Qualysis system, including twelve Oqus cameras [39] and sixty-one retro-reflective markers. These markers were placed on different body landmarks as illustrated in Fig 2. The subjects were asked to wear tight-fitting shorts and upper body garment to allow the proper placement of the markers.

The RGB-D camera was mounted on a tripod, and connected to a portable computer. The height of the camera, i.e., the distance between its base bottom and the floor, was of 1 m. The tilt angle in relation to the horizontal plane (perpendicular to the gravity force) was of −5°. For this setup, the practical range, i.e., the range for which the camera is able to track all body joints, is of 2.9 m (1.5 m to 4.4 m from the sensor).

### Experimental protocol

The experimental protocol included three different tasks (T1, T2 and T3), which are described in detail in Table 2. Both T1 and T2 consist of walking towards and away from the Kinect sensor, only differing in the distances covered (7 m for T1, and 5 m for T2), and the distance from the sensor at which the subjects turns around (outside the Kinect’s practical depth range for T1, and inside the range for T2). Task T3 is similar to T1, but it also includes the activities of standing still and marching in a military style (see Table 2). The latter activities were included in the protocol, since in a real situation the subject may not always perform the defined gait task precisely.

**Table 2:** Description of the tasks performed during the experiment, and associated number of trials.

Task name	Description	No. of trials
**T1**	Walk towards the Kinect for 7 m; turn around at 1.2 m from the sensor (outside its practical depth range); walk away from the sensor for another 7 m.	10
**T2**	Walk towards the Kinect for 5 m; turn around at 3.2 m from the sensor (within its practical depth range); walk away from the sensor for another 5 m.	5
**T3**	Walk towards the Kinect for 5 m; stop at 3.2 m from the sensor and stand still for 5 seconds; march in a military style (i.e., move the left and right feet up and down alternately, three times each, while standing in the same place); walk towards the sensor for 2 m; turn around at 1.2 m from the sensor; walk away from the sensor, repeating the same activities as for walking towards the sensor.	5

Each subject carried out the number of trials per task indicated in Table 2. For all tasks, walking was performed at a self-selected pace.

### Data acquisition and pre-processing

**Fig 3:**
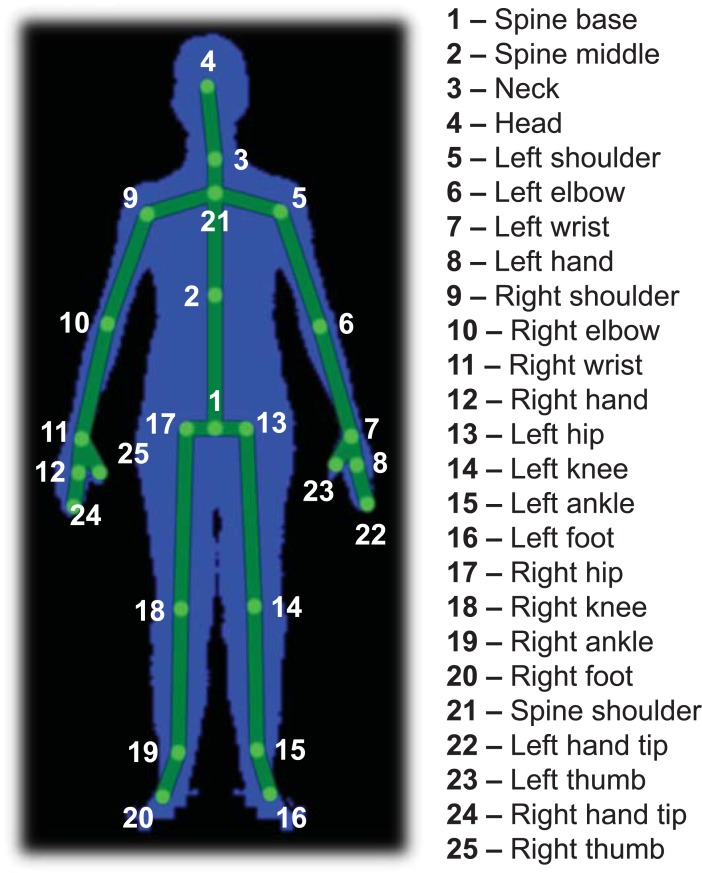
Body joints tracked by the Kinect v2.

Data provided by the Kinect were acquired at 30Hz, using our *KiT* software application [40]. Data provided by the Qualysis system were acquired at 200 Hz. The Kinect data included infrared, depth and 3-D body joint data. Each frame of the latter includes the 3-D position of the joints illustrated in Fig 3. The Qualysis data included the markers’ 3-D position, which was measured with an accuracy of at least 0.6 mm.

For activity recognition, we manually selected the time intervals corresponding to the different activities (walking, standing, and marching), using our *KiMA* software application [40]. We also distinguished between two different postures: facing the sensor (front) or facing away from the sensor (back). The following six activities were considered:

- Walking towards the sensor (WF);
- Walking away from the sensor (WB);
- Standing while facing the sensor (SF);
- Standing while facing away from the sensor (SB);
- Marching in a military style while facing the sensor (MF);
- Marching in a military style while facing away from the sensor (MB).

The number of frames and duration of the selected data for each and all activities are presented in Table 3, considering all subjects. The mean and standard deviation values per subject are also shown.

**Table 3:** Number of frames and duration of the selected Kinect data, for each and all activities, considering all subjects, as well as each subject (mean and standard deviation).

Activity	No. of frames		Duration (min)	
	All subjects	Per subject	All subjects	Per subject
**WF**	22,439	1,122 ± 96	12.3	0.6 ± 0.1
**WB**	23,899	1,195 ± 104	13.1	0.7 ± 0.1
**SF**	12,558	628 ± 60	6.9	0.3 ± 0.0
**SB**	12,198	610 ± 67	6.7	0.3 ± 0.0
**MF**	13,280	664 ± 44	7.3	0.4 ± 0.0
**MB**	13,443	672 ± 42	7.4	0.4 ± 0.0
**All**	97,817	4,891 ± 314	53.7	2.7 ± 0.2

We took into account two different postures of the subject (facing the sensor or facing away from it), because the Kinect itself does not distinguish between them. The Kinect assumes that the subject is always facing the sensor, which means that when the subject is facing away from it, the left and right joints are interchanged. Therefore, the ability to distinguish between the two postures is important for gait analysis, when the data includes both walking towards and away from the camera.

To evaluate our method for detecting heel strikes, we used the data corresponding to the walking activity performed during task T1. Moreover, it was necessary to synchronize the Kinect and Qualysis data. This synchronization was performed by using the instant of the following action, included in the protocol, as a common time reference: dropping an extra marker on the floor before each trial. The instant was identified based on the infrared images in the case of the Kinect, and on the y-coordinate of the extra marker’s position for the Qualysis.

The synchronization process was performed and validated for each trial. For the validation, we firstly resampled the Kinect data to a fixed frame rate of 200 Hz. Then, we obtained the signal corresponding to the sum of the three coordinates of the left ankle’s position, for both Kinect and Qualysis. The Pearson’s correlation coefficient for the two signals (*r*_la_) was computed. The same procedure was followed to obtain the correlation coefficient for the right ankle (*r*_ra_). To ensure that only data with good synchronization between systems were used for the algorithm evaluation, the trials for which *r*_la_ or *r*_ra_ was lower than 0.9 were not taken into account.

The number of actual heel strikes and gait cycles performed by all subjects is indicated in Table 4, for the analyzed trials. The mean and standard deviation values per subject and per trial are also included.

**Table 4:** Number of actual heel strikes and gait cycles performed by all subjects, per subject, and per trial, when considering all analyzed trials of task T1.

Event	Side	All subjects and trials		Per subject		Per trial	
		WF	WB	WF	WB	WF	WB
**Heel strike**	**Left**	453	402	22.6 ± 3.4	20.1 ± 2.5	2.4 ± 0.5	2.1 ± 0.3
	**Right**	407	392	20.4 ± 2.4	19.6 ± 3.6	2.1 ± 0.3	2.0 ± 0.5
	**Both**	860	794	43.0 ± 4.3	39.7 ± 5.2	4.5 ± 0.6	4.1 ± 0.6
**Gait cycle**	**Left**	261	207	13.1 ± 3.3	10.4 ± 2.3	1.4 ± 0.5	1.1 ± 0.3
	**Right**	215	197	10.8 ± 2.2	9.8 ± 3.6	1.1 ± 0.3	1.0 ± 0.5
	**Both**	476	404	23.8 ± 4.0	20.2 ± 5.0	2.5 ± 0.6	2.1 ± 0.6

The results per subject and per trial are presented as mean and standard deviation values. All results are indicated for the left, right, and both heel strikes/gait cycles, as well as for walking towards and away from the sensor (WF and WB, respectively).

### Computation of kinematic measures

With the aim of activity recognition, the thirty-eight kinematic measures described in Table 5 were extracted, for each Kinect data frame. These measures include the velocity of each tracked joint, which corresponds to the magnitude of the associated velocity vector. The velocity of trunk joints (i.e., head, neck, spine middle, spine base, and spine shoulder) may help distinguish between walking and standing/marching, while the velocity of the remaining joints may help distinguish between standing and walking/marching. We also considered the z-axis velocity (i.e., the z-axis component of the velocity vector) of the trunk joints, since it was expected to help in the distinction between walking towards and away from the sensor.

**Table 5:** Kinematic measures computed over the 3-D body joint data for activity recognition, and corresponding equations.

Measure	Joints	Equation
**Velocity**	Head, neck, spine middle, spine base, and spine shoulder	(1)
	Shoulder, elbow, wrist, hand, hip, knee, ankle, foot, hand tip, and thumb^a^	
**Z-axis velocity**	Head, neck, spine middle, spine base, and spine shoulder	(2)
**Distance between symmetrical joints**	Left and right feet, ankles, knees, hands, wrists, elbows, hand tips, and thumbs	(3)
**Angle**	Neck, spine shoulder, and spine middle^b^	(4)
	Knee, and elbow^c^ ^a^	
**YZ-plane angle**	Neck, spine shoulder, and spine middle^b^	(5)
	Knee, and elbow^c^ ^a^	

^a^ corresponds to the mean of left and right joint measures, where the measure for each side was computed using the indicated equation.

^b^ considering the head, neck, and spine shoulder joints, the neck, spine shoulder, and spine middle joints, and the spine shoulder, spine middle, and spine base joints, respectively.

^c^ considering the hip, knee and ankle joints, and the wrist, elbow and shoulder joints, respectively.

We also computed the distance between symmetrical joints (e.g., left and right hands), since it varies for the walking and marching activities, while it is expected to not vary significantly for standing. Moreover, the distance between ankles, together with the velocity of each ankle, is used for heel strike detection.

Other extracted measure is the angle at given joints of the trunk, which provides information regarding posture. For the body limbs, we computed the angle at the elbows and knees, since these should have the largest variation during walking. We also considered the YZ-plane angle (considers only the y- and z- components of the joints’ position), since its value indicates the direction of the angle in relation to the XY-plane, and can be useful for detecting weather the subject is facing the sensor or facing away from it.

$$\mathrm{velocity}=\sqrt{v_{x}^{2}+v_{y}^{2}+v_{z}^{2}}\approx \sqrt{\frac{\Delta x^{2}+\Delta y^{2}+\Delta z^{2}}{\Delta t^{2}}}$$ (1)

$$v_{z}\approx \frac{\Delta z}{\Delta t}$$ (2)

$$\mathrm{distance}=\left\|\vec{P_{\mathrm{left}}P_{\mathrm{right}}}\right\|$$ (3)

$$\mathrm{angle}=\arccos\left(\frac{\vec{P_{2}P_{1}}\cdot \vec{P_{2}P_{3}}}{\left\|\vec{P_{2}P_{1}}\right\|\times \left\|\vec{P_{2}P_{3}}\right\|}\right)$$ (4)

$${\mathrm{angle}}_{\mathrm{yz}}=\arctan\;\left(\frac{y_{1}z_{2}-z_{1}y_{2}}{y_{1}y_{2}+z_{1}z_{2}}\right),\;\vec{P_{2}P_{1}}=\left(x_{1},y_{1},z_{1}\right)\;\mathrm{and}\;\vec{P_{2}P_{3}}=\left(x_{2},y_{2},z_{2}\right)$$ (5)

In (1), *v*_*x*_ is the x-axis component of the velocity vector for a given joint, and Δ*x* is the difference between the x-coordinate of the joint position considering two consecutive frames. Similar notations are used for the y- and z-axis. Δ*t* is the time elapsed between two consecutive frames. In (3), *P*_left_ and *P*_right_ refer to the 3-D position of the considered left and right joint, respectively. In (3) and (4), $\vec{P_{i}P_{j}}$ is the 3-D vector defined by positions *P*_*i*_ and *P*_*j*_. In (4) and (5), *P*_1_, *P*_2_ and *P*_3_ correspond to the 3-D position of three different joints.

### Activity recognition

**Fig 4:**
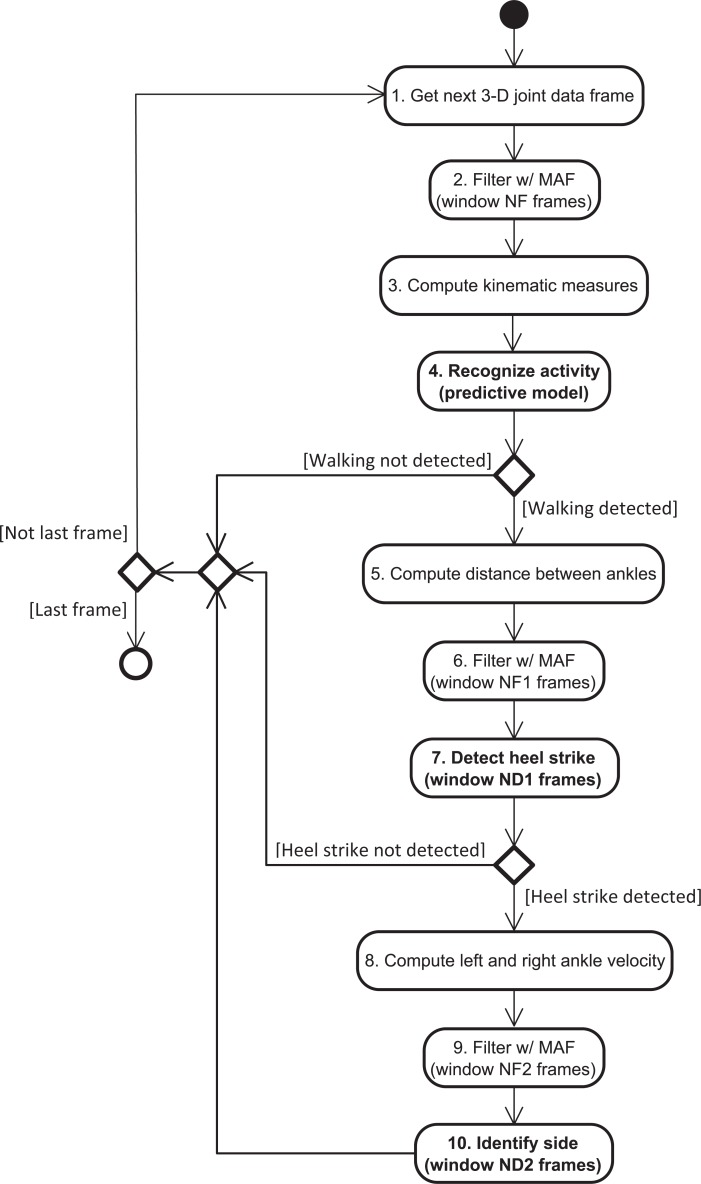
Solution for gait cycle detection, including activity recognition and heel strike estimation. MAF stands for moving average filter.

Our solution detects the time intervals corresponding to the walking activity by recognizing the following six activities or classes: walking front and back, standing front and back, marching front and back - WF, WB, SF, SB, MF, MB. The activity recognition is carried out by using a predictive model, and the measures included in Table 5. These measures are obtained from 3-D body joint data that were processed by using a moving average filter with a window size of *NF* frames: for each axis, the filtered value of sample *i* is the mean value of all samples within the window (centered on sample *i*). These steps are illustrated in Fig 4 by the second to fourth blocks.

To obtain the predictive model for activity recognition, we explored the following machine learning algorithms: k-nearest neighbors, classification tree, random forest, support vector machines, multilayer perceptron, and multilayer perceptron ensemble. For each algorithm, the performance of the corresponding model was estimated by using a stratified 10-fold cross-validation approach [41]. We then built a final model using the algorithm that led to the model with best trade-off between the considered metrics (accuracy, F_1_ score, training and prediction time).

All predictive models were trained, validated and tested in the R environment [42], using the *rminer* package [43]. The value of the window size *NF* was chosen by considering the odd integer values in the range [3, 11], and selecting the one that led to the highest mean overall accuracy for all algorithms.

The dataset used to compare the algorithms and obtain the final model included 35,010 frames or instances, and 38 kinematic measures, corresponding to fifteen subjects (randomly chosen from the twenty subjects that participated in the study). The number of instances corresponds to 389 frames per subject and activity, which were selected from the original dataset by performing random under-sampling (without replacement). The number of selected frames per subject and activity corresponds to the minimum number of frames, when taking into account all subject-activity combinations in the original dataset. This was done in order to have a balanced dataset. The final model was tested over a dataset corresponding to the five subjects whose data were not used for model training (“never seen” subjects), which includes 23,412 instances.

#### Machine learning algorithms

In our study, we used a weighted version of the *k*-nearest neighbors (*k*-NN) algorithm [44, 45]. The number of considered nearest neighbors (*k*) was 7, the distance between two instances was computed using the Euclidean distance, and the weight of the *k* nearest neighbors was obtained using the "optimal" kernel function [46].

For the classification tree, we used an implementation [47, 48] of the recursive partitioning method for building classification and regression trees (CART) [49]. The Gini index was used to compute the impurity of a node. The class frequency in the training set was considered as the class prior probability. In the used implementation, a split is not attempted if the node does not have a minimum of 20 instances, or if the split does not lead to an improvement by a factor of 0.01 (complexity parameter) [47, 48].

For the random forest algorithm, we used an implementation [50] of Breiman's random forest [51]. The number of grown trees was 500, and the size of the feature subset selected for each node was $\left\lfloor \sqrt{n}\right\rfloor$, where *n* is the number of features in the training set.

For the support vector machine (SVM) algorithm, we used the C-SVM formulation, with a cost parameter value of 1. For the kernel function, we used the Gaussian radial basis function *K*(**x**,**x**') = exp(−*σ*‖**x**−**x**'‖^2^), where **x** and **x'** are two instances. In the used implementation of SVM [52, 53], the value of parameter *σ* is the median of the 0.1 and 0.9 quantile of the ‖**x**−**x**'‖^2^ statistics for a sample of the training set. The optimization problem is solved by relying on the sequential minimal optimization (SMO) algorithm [54]. For multi-class problems, the one-against-one approach is used.

For the multilayer perceptron (MLP) algorithm, we used the implementation described in [55]. The activation function of the hidden neurons is the logistic function. For multi-class problems, the output layer has a linear neuron per class. The search for the best set of weights between nodes is carried out by the BFGS (Broyden–Fletcher–Goldfarb–Shanno) algorithm, which minimizes a fitting criterion (maximum likelihood, in the case of classification) [43, 55]. It is stopped when the error slope approaches zero or after a maximum number of iterations (we considered 100 iterations). The number of hidden neurons was set to 10.

Since the training of a MLP model is not optimal, to avoid the dependence of the final solution on the choice of starting weights (chosen at random), a given number of different MLP models are built [43] (we considered three models). Then, the one with the lowest value of the fitting criterion is selected. In the case of the MLP ensemble (MLPE), all built MLPs are used, and the output is the mean of the individual predictions.

#### Model evaluation

The performance of a given model was evaluated using the following metrics: accuracy and F_1_ score. The class and overall accuracy were computed using (6) and (7), respectively. The class and overall F_1_ score were computed using (8) and (9), respectively. In (9), *C* is the number of classes, F_1_(*c*_*i*_) is the F1 score for class *c*_*i*_, and prev(*c*_*i*_) is the prevalence of class *c*_*i*_ given by $N_{c_{i}}/N$, where *N* and $N_{c_{i}}$ are the number of total instances and instances of class *c*_*i*_, respectively, in the testing set. The value of both the accuracy and F_1_ score ranges between 0% and 100% inclusive, where a higher value is better.

$$\mathrm{Class}\;\mathrm{accuracy}\left(\%\right)=\frac{\mathrm{TP}+\mathrm{TN}}{\mathrm{TP}+\mathrm{TN}+\mathrm{FP}+\mathrm{FN}}\times 100$$ (6)

$$\mathrm{Overall}\;\mathrm{accuracy}\left(\%\right)=\frac{\mathrm{number}\;\mathrm{of}\;\mathrm{correctly}\;\mathrm{classified}\;\mathrm{instances}}{\mathrm{number}\;\mathrm{of}\;\mathrm{total}\;\mathrm{instances}}\times 100$$ (7)

$$\mathrm{Class}\;F_{1}\;\mathrm{score}=2\times \frac{\mathrm{class}\;\mathrm{precision}\times \mathrm{class}\;\mathrm{sensitivity}}{\mathrm{class}\;\mathrm{precision}+\mathrm{class}\;\mathrm{sensitivity}}$$ (8)

$$\mathrm{Overall}\;F_{1}\;\mathrm{score}=\sum_{i=1}^{C}F_{1}\left(c_{i}\right).\mathrm{prev}\left(c_{i}\right)$$ (9)

$$\mathrm{Class}\;\mathrm{sensitivity}\left(\%\right)=\frac{\mathrm{TP}}{\mathrm{TP}+\mathrm{FN}}\times 100$$ (10)

$$\mathrm{Class}\;\mathrm{precision}\left(\%\right)=\frac{\mathrm{TP}}{\mathrm{TP}+\mathrm{FP}}\times 100$$ (11)

In (6), (10) and (11), TP, TN, FP and FN correspond to:

- True positives (TP): the number of instances correctly classified as belonging to the considered class;
- True negatives (TN): the number of instances correctly classified as belonging to a class other than the one considered;
- False positives (FP): the number of instances incorrectly classified as belonging to the considered class;
- False negatives (FN): the number of instances incorrectly classified as belonging to a class other than the one considered.

As our objective is to develop an online solution for gait cycle detection, the model should be able to predict the activity in the shortest possible amount of time, besides achieving a high accuracy and F_1_ score. A low training time is also desirable. Therefore, we also considered the training time, as well as the prediction time for a single data frame, and for 1 min of data (≈1,800 frames).

### Gait cycle detection

For detecting the gait cycles that occur during the walking activity, we implemented an algorithm that estimates the instants corresponding to left and right heel strikes. To develop this algorithm, we took into account only the data corresponding to task T1, since it is the task for which activities WF and WB have the longest duration, including a greater number of consecutive heel strikes and consequently more gait cycles. The algorithm implementation and evaluation were performed in Matlab (version R2015a).

#### Algorithm implementation

For the detection of heel strikes, we firstly explored the kinematic measures described in Table 6, which were extracted from the Qualysis data (ground truth). The foot velocity signals were processed using a fourth order zero lag low-pass Butterworth filter with a cut-off frequency of 8 Hz. The latter value was chosen taking into account the frequency content of the signals.

**Table 6:** Kinematic measures computed over the Qualysis 3-D data, and corresponding equations.

Measure	Equation	Joint position computed from Qualysis markers^a^
**Vertical velocity of left foot**	*v*_*y*_ ≈ Δ*y*/Δ*t*	*y* = ((*y*_LLA_ + *y*_LMA_)/2 + *y*_LFM2_)/2
**Vertical velocity of right foot**		*y* = ((*y*_RLA_ + *y*_RMA_)/2 + *y*_RFM2_)/2
**Velocity of left foot**	(1)	*P* = ((*P*_LLA_ + *P*_LMA_)/2 + *P*_LFM2_)/2
**Velocity of right foot**		*P* = ((*P*_RLA_ + *P*_RMA_)/2 + *P*_RFM2_)/2
**Distance between ankles**	(3)	*P*_left_ = (*P*_LLA_ + *P*_LMA_)/2, *P*_right_ = (*P*_RLA_ + *P*_RMA_)/2
**Distance between knees**		*P*_left_ = (*P*_LLK_ + *P*_LMK_)/2, *P*_right_ = (*P*_RLK_ + *P*_RMK_)/2

The computation of the joint(s) position based on the Qualysis markers’ position is indicated for each measure. The markers’ placement is (Fig 2): LLA and LMA on the left ankle; RLA and RMA on the right ankle; LFM2 on the left foot; RFM2 on the right foot; LLK and LMK on the left knee; RLK and RMK on the right knee.

^a^ *P*_*name*_ and *y*_*name*_ correspond to the 3-D position and y-coordinate of the position of marker *name*, respectively.

**Fig 5:**
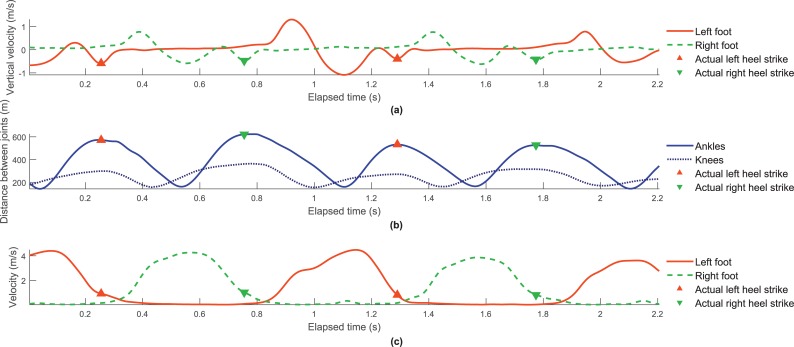
Measures computed over Qualysis data acquired from a given subject while walking towards the Kinect. (a) Filtered left and right foot vertical velocity, versus the elapsed time. (b) Distance between ankles, and between knees, versus the elapsed time. (c) Filtered left and right ankle velocity, versus the elapsed time. The actual left and right heel strikes are indicated in each plot.

Following the findings of O’Connor et al. [25], we identified the actual heel strike instants using the feet vertical velocity. Fig 5A shows an example of the left and right foot vertical velocity versus the elapsed time, and the associated heel strike events, for a given subject and WF trial.

The Kinect data can be rather noisy, making it more difficult to detect heel strikes based on the feet vertical velocity. Therefore, we considered identifying the heel strike instants by finding the local maxima of the knee distance signal, similarly to the method proposed by Auvinet et al. [30]. However, we verified visually that in most cases the actual heel strike instants are closer to the instants corresponding to the local maxima of the ankle distance, when compared with the knee distance, as can be seen from Fig 5B. This figure shows both the knee and ankle distance versus the elapsed time, as well as the actual heel strike instants, for the same subject and trial as Fig 5A.

Therefore, our solution estimates heel strike instants by finding the local maxima of the distance between ankles. A sample *i* is identified as a heel strike if the associated ankle distance is maximum, when considering a window with size of *ND1* samples (centered on sample *i*). Prior to this processing, a moving average filter with a window size of *NF1* samples was applied to the used data. These operations are represented in Fig 4 by the fifth to seventh blocks.

To detect each gait cycle, it is further necessary to identify the side (left or right) associated with each detected heel strike. To achieve this, we investigated the possibility of using the left and right ankle velocity. As expected, when a left/right heel strike occurs, the velocity of the left/right ankle is decreasing, while the velocity of the right/left ankle is increasing. This can be seen in Fig 5C, which shows the left and right foot velocity versus the elapsed time, and the actual heel strike instants, for the same subject and trial as Fig 5A and 5B.

Our algorithm identifies the side associated with a given heel strike by using the following decision rule:

$$\Delta v_{\mathrm{left}}-\Delta v_{\mathrm{right}}\{\begin{array}{l} <0\;\to \mathrm{left}\;\mathrm{heel}\;\mathrm{strike}\\ =0\;\to \mathrm{undefined}\\ >0\;\to \mathrm{right}\;\mathrm{heel}\;\mathrm{strike} \end{array}$$

In this expression, Δ*v*_*side*_ = *v*_*side*_ (*i* + *N*) − *v*_*side*_(*i* − *N*), where *side* is left or right, *v*_*side*_(*k*) is the ankle velocity for sample *k*, *i* is the sample corresponding to the detected heel strike, and *N* = (*ND2*−1)/2, where *ND2* is the size of the processing window. The ankle velocity data is previously processed using a moving average filter with a window size of *NF2* samples. The operations related to side detection are illustrated in Fig 4 by the eighth to tenth blocks.

To find the best values for *NF1* and *ND1*, we considered the odd integer values in the range [1, 7] and [3, 15], respectively. From all window size pairs, we selected the one that we considered to have the best trade-off between precision, sensitivity, and mean absolute error for heel strike instant estimation. For the chosen *NF1* and *ND1* values, we then performed a similar selection for the window sizes *NF2* and *ND2* used for side identification. In this case, we chose the pair of values that led to the highest mean of precision and sensitivity, where the precision/sensitivity value is the mean between the left and right sides.

#### Evaluation of gait cycle detection

The evaluation of the performance of our gait cycle detection algorithm was based on the true and absolute estimation errors for each heel strike instant, using (12) and (13), respectively. To compare with the results of other studies, we also we also computed spatiotemporal gait parameters (stride duration and length, step duration and length, and gait speed) and their associated estimation errors. The stride and step durations correspond to the time interval between two consecutive heel strikes of the same and opposite sides, respectively. The stride and step lengths are the distance between the position of the ankle joint at the instants corresponding to the beginning and end of the stride and step, respectively. The gait speed is the stride length divided by stride duration.

trueerror=estimatedvalue−actualvalue (12)

absoluteerror=|estimatedvalue−actualvalue| (13)

To evaluate our algorithm, we also computed the sensitivity (10) and precision (11) regarding the detection of heel strikes, and the identification of the side associated with each detected heel strike.

For the detection of heel strikes, TP, FN and FP correspond to:

- True positives (TP): number of estimated heel strike instants that fall in time interval ]*t*_*i*_ − 0.4,*t*_*i*_ + 0.4[ s, where *t*_*i*_ is the instant of an actual heel strike *i*;
- False negatives (FN): number of actual heel strikes for which no heel strike is detected in time interval ]*t*_*i*_ − 0.4,*t*_*i*_ + 0.4[ s;
- False positives (FP): number of estimated heel strike instants that are not within any time interval ]*t*_*i*_ − 0.4,*t*_*i*_ + 0.4[ s.

If more than one heel strike is detected within a given time interval ]*t*_*i*_ − 0.4,*t*_*i*_ + 0.4[, only the first one is considered as true positive (the remaining are false positives). The value of 0.4 s used above corresponds to approximately half of 0.87 s, which is the minimum of the approximate range of stride duration for free-speed walking performed by normal adults [1].

For identifying the heel strike side, when considering the left/right side as the positive class, TP, FP and FN correspond to:

- True positives (TP): number of actual left/right heel strikes correctly identified as left/right;
- False positives (FP): number of actual right/left heel strikes incorrectly identified as left/right;
- False negatives (FN): number of actual left/right heel strikes incorrectly identified as right/left.

## Results

### Activity recognition

The overall accuracy and F_1_ score achieved by the models built using different machine learning algorithms are presented in Table 7. The window size used for filtering the 3-D joint data was of 17 frames, since it led to the highest mean overall accuracy considering all algorithms.

**Table 7:** Performance results achieved by the models built with different machine learning algorithms.

Metric		Machine learning algorithms					
		k-NN	Decision tree	Random forest	SVM	MLP	MLPE
**Overall accuracy (%)**		98.6	95.1	98.6	98.3	98.3	98.4
**Overall F**_**1**_ **score (%)**							
**Training time (min)**		0.0 ± 0.0	0.1 ± 0.0	3.3 ± 0.4	0.5 ± 0.0	1.7 ± 0.1	1.5 ± 0.1
**Prediction time**	**1 frame (ms)**	349 ± 165	3 ± 7	67 ± 44	10 ± 10	5 ± 8	12 ± 9
	**1,800 frames (min)**	10.5 ± 4.9	0.1 ± 0.2	2.0 ± 1.3	0.3 ± 0.3	0.2 ± 0.2	0.4 ± 0.3

The models’ performance results include the overall accuracy and overall F_1_ score, as well as the mean and standard deviation values for the training time when considering five runs, and the prediction time for a single frame and for 1,800 frames (≈1 min of data).

The prediction time per frame, as well as for 1,800 frames (≈ 1 min of data), for each model is also presented in Table 7. The same table includes the models’ training time (mean and standard deviation), when considering five runs. These results were obtained on a computer with an i7-4600U CPU (dual-core, 2.1 GHz), and 8 GB RAM.

From the explored algorithms, we selected the MLP algorithm, since the associated model presents the best trade-off between all considered metrics. Table 8 shows the accuracy and F_1_ score achieved by the final MLP model, when performing activity recognition over the dataset of five “never seen” subjects (23,482 instances).

**Table 8:** Accuracy and F_1_ score achieved by the final MLP model when predicting the activity over a dataset of five “never seen” subjects.

Activity	Accuracy (%)	F_1_ score (%)
**WF**	99.3	98.4
**WB**	98.7	97.4
**SF**	99.6	98.4
**SB**	99.3	97.3
**MF**	99.5	98.2
**MB**	99.0	96.5
**Overall**	97.7	97.7

The accuracy and F_1_ score for each activity or class were obtained using (6) and (8), respectively. The overall values of these metrics were computed using (7) and (9), respectively.

The results of activity recognition for a trial of each considered task (T1, T2, and T3), performed by a given subject, can be seen in S1 Video. In this video, our *KiMA* application [40] is used to show the acquired data, as well as the different recognized activities, which are represented as “events”.

### Gait cycle detection

The gait cycle detection algorithm uses different window sizes for ankle distance signal filtering (*NF1*), heel strike detection (*ND1*), ankle velocity signal filtering (*NF2*), and heel strike side identification (*ND2*). The used window sizes, as well as the corresponding precision and sensitivity results, are included in Table 9. Table 10 presents the mean and standard deviation of the true and absolute errors for estimating heel strike instants and gait parameters, when considering all subjects, trials, and detected heel strikes/gait cycles.

**Table 9:** Window sizes used for gait cycle detection, as well as the corresponding achieved precision and sensitivity, for WF and WB trials.

			Walking activity	
			WF	WB
**Window size** **(no. of frames)**	**Ankle distance filtering (*NF1*)**		5	5
	**Heel strike detection (*ND1*)**		5	9
	**Ankle velocity filtering (*NF2*)**		3	3
	**Heel strike side identification (*ND2*)**		9	11
**Precision** **(%)**	**Heel strike detection**		99.4	99.9
	**Side identification**	**Left**	99.8	100.0
		**Right**	99.7	100.0
**Sensitivity** **(%)**	**Heel strike detection**		98.1	98.1
	**Side identification**	**Left**	99.8	100.0
		**Right**	99.7	100.0

**Table 10:** Mean and standard deviation of the true and absolute errors for estimating heel strike instants and gait parameters, when considering all subjects, trials, and detected heel strikes/gait cycles, for WF and WB trials.

		True error		Absolute error	
		WF	WB	WF	WB
**Heel strike instant (ms)**	**Left**	14.8 ± 29.2	11.9 ± 23.6	21.9 ± 24.3	20.8 ± 16.2
	**Right**	14.4 ± 19.3	12.3 ± 21.9	19.2 ± 14.6	19.5 ± 15.7
	**Both**	14.6 ± 25.0	12.1 ± 22.7	20.6 ± 20.3	20.2 ± 16.0
**Stride duration (ms)**	**Left**	2.7 ± 35.4	2.4 ± 23.8	20.4 ± 29.1	18.3 ± 15.3
	**Right**	−1.3 ± 17.7	1.7 ± 24.9	13.3 ± 11.7	18.0 ± 17.3
	**Both**	0.9 ± 28.9	2.0 ± 24.3	17.2 ± 23.2	18.1 ± 16.3
**Step duration (ms)**	**Left**	0.4 ± 25.4	3.5 ± 23.6	19.1 ± 16.7	18.3 ± 15.3
	**Right**	1.6 ± 19.9	1.8 ± 23.8	15.3 ± 12.8	17.6 ± 16.0
	**Both**	0.9 ± 23.1	2.7 ± 23.7	17.4 ± 15.1	18.0 ± 15.6
**Stride length (cm)**	**Left**	1.2 ± 1.7	−0.2 ± 3.3	1.7 ± 1.3	2.5 ± 2.2
	**Right**	0.6 ± 1.3	0.0 ± 3.8	1.1 ± 1.0	2.9 ± 2.5
	**Both**	0.9 ± 1.6	−0.1 ± 3.6	1.4 ± 1.2	2.7 ± 2.4
**Step length (cm)**	**Left**	−3.3 ± 4.9	−4.2 ± 5.5	4.2 ± 4.2	5.6 ± 4.0
	**Right**	−2.2 ± 3.5	−4.1 ± 5.5	3.3 ± 2.5	5.4 ± 4.1
	**Both**	−2.8 ± 4.4	−4.1 ± 5.5	3.8 ± 3.5	5.5 ± 4.0
**Gait speed (m/s)**	**Left**	0.010 ± 0.032	−0.005 ± 0.038	0.017 ± 0.029	0.028 ± 0.025
	**Right**	0.007 ± 0.014	−0.002 ± 0.037	0.012 ± 0.010	0.029 ± 0.023
	**Both**	0.009 ± 0.026	−0.003 ± 0.037	0.015 ± 0.023	0.029 ± 0.024

**Fig 6:**
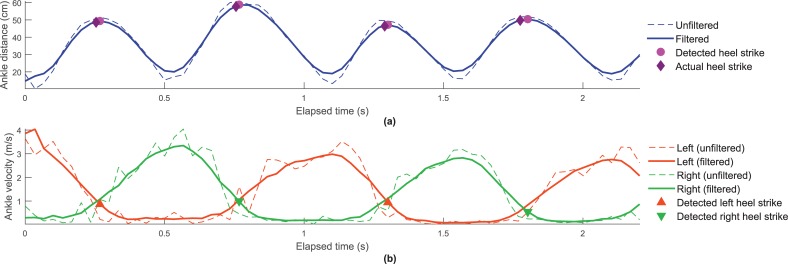
Measures computed from unfiltered and filtered Kinect data, for the same subject and WF trial of Fig 5. (a) Distance between ankles versus the elapsed time, including the indication of the estimated and actual heel strike instants. (b) Velocity of left and right ankles versus the elapsed time, including the indication of the detected left and right heel strikes.

Fig 6A presents the ankle distance computed from both unfiltered and filtered Kinect data, versus the elapsed time, for the same subject and trial of Fig 5. It also shows the heel strike instants estimated by our algorithm, as well as the actual heel strikes instants. The identification of the side (left or right) associated with the detected heel strikes is indicated in Fig 6B. This figure is similar to Fig 6A, but it presents the left and right ankle velocity.

The results of heel strike detection for a trial of each considered task (T1, T2, and T3), performed by a given subject, can be seen in S1 Video. In this video, the detected heel strikes as shown “labels” in our *KiMA* application [40].

## Discussion

### Activity recognition

From the different obtained models for activity recognition, the decision tree model achieved the poorest performance, when considering the overall accuracy and F_1_ score (see Table 7). All other models achieved similar predictive results (overall accuracy and F_1_ score between 98.3% and 98.6%). However, the k-NN and random forest models are not suitable for online activity recognition, since they take too long to predict the activity for 1 minute of data (mean prediction time of 11 and 2 min, respectively – see Table 7). From the remaining algorithms, we chose the MLP, since the corresponding model has a lower mean prediction time per frame when compared with the MLPE and SVM models.

When using the final MLP model over a dataset corresponding to five never seen subjects, it achieved accuracy and F_1_ score values greater than 98.7% and 96.5%, respectively (Table 8). When taking into account the two different postures, i.e. facing the sensor (WF) and facing away from the sensor (WB), recognition is better for the first.

### Gait cycle detection

The precision and sensitivity values achieved by our gait cycle detection algorithm are high (≥ 99.4% and ≥ 98.1%, respectively – Table 9) for both WF and WB activities. A high precision for heel strike detection is important, since false positives lead to the incorrect computation of gait parameters. A high sensitivity is also desirable, since it means that a greater number of actual gait cycles are detected for the same acquired data. This is useful when we wish to perform gait analysis based on a minimum number of gait cycles, because it allows saving time during the process of data acquisition and analysis.

The heel strike instants and gait parameters tend to be overestimated (positive mean true errors) by our algorithm, with the exception of the step length (WF and WB), stride length (WB), and gait speed (WB). The true and absolute errors for heel strike instants are similar when comparing WF and WB (only slightly better for WB overall). For the gait parameters, there are also no considerable differences between the two types of walking. So, WF and WB data can both be used for gait analysis with a similar degree of confidence. This information is useful, since it allows maximizing the number of gait cycles acquired per gait task repetition, consequently reducing the time required for performing gait analysis of a given subject.

To the best of our knowledge, all studies on gait cycle detection using Kinect relied on the first version of the sensor (Kinect v1) [17, 18, 21, 30, 31], with the exception of one that used its second version [38]. In the latter study, the error of estimating gait events/parameters was not reported. Therefore, it is not possible to compare their study with ours. A direct comparison between our results and the ones obtained using Kinect v1 is also not possible, not only due to the use of a different version of the Kinect, but also because different experimental setups and/or protocols were used (e.g., treadmill instead of overground walking, different configurations of the Kinect).

Nevertheless, it is interesting to see how our results stand in relation to the results reported in other contributions. For heel strike estimation, our algorithm obtained a true error of 14.6 ± 25.0 ms (WF), while Auvinet et al. reported a true error of 17 ± 24 ms [30] (treadmill walking while facing the Kinect). Regarding the stride and step duration, we achieved true errors of 0.9 ± 28.9 ms and 0.9 ± 23.1 ms (WF), which is quite lower than the one reported by Clark et al.: −200 ± 66 ms and −170 ± 71 ms (walking towards the sensor) [21]. Our mean true error for stride duration is similar to the one presented by Auvinet et al. (0 ± 12 ms [30]).

For the remaining parameters (stride length, step length, and gait speed) we obtained the following true errors for WF trials: 0.9 ± 1.6 cm, −2.8 ± 4.4 cm, and 0.009 ± 0.026 m/s. These results are similar to those reported by Clark et al.: −0.4 ± 2.9 cm, 1.2 ± 1.6 cm, and −0.010 ± 0.038 m/s, respectively [21].

## Conclusions

We presented a system for fully automatic gait analysis using a single low-cost, portable and markerless RGB-D camera, namely the Kinect v2. Our system includes a solution that detects the walking activity, and identifies gait cycles only when walking is detected. This solution allows to carry out gait analysis without any manual and/or external intervention (besides starting and stopping the data acquisition). Moreover, it can be used online, allowing to verify the number of gait cycles detected up to a given instant during a data acquisition. This ability is important if we wish to perform gait analysis based on a given minimum number of gait cycles.

Our solution can be used with any other RGB-D camera that provides the 3-D position of the main body joints. For each detected gait cycle, the system extracts several gait parameters (e.g., stride and step duration and length, and gait speed). These parameters can provide useful information in multiple contexts, such as sports, biometric identification, and healthcare.

The detection of the walking activity is carried out by a predictive model that recognizes three different activities: walking, standing, and marching. Therefore, this model may be used to support the assessment of leg agility and posture, besides gait analysis. Furthermore, the model distinguishes between two different positions of the subject: facing the sensor, and facing away from the sensor. This ability is useful for performing gait analysis when the data includes both walking towards and away from the camera, since the Kinect itself does not distinguish between these two situations. The model was built using the MLP algorithm and data acquired from fifteen subjects. It achieved an overall accuracy and F_1_ score of 98%, when used over data corresponding to five “never seen” subjects.

For gait cycle detection, we implemented an algorithm that estimates heel strike instants based on the distance between ankles, and identifies the associated side (left or right) based on the velocity of the left and right ankles. The algorithm was evaluated over data corresponding to ten gait trials from twenty subjects. The obtained results show that the data acquired while the subject is walking both towards and away from the sensor (WF and WB) can be used for gait analysis with a similar degree of confidence (e.g., true errors for heel strike instant and stride duration of 15 ± 25 ms and 1 ± 29 ms for WF, and of 12 ± 23 ms and 2 ± 24 ms for WB).

As future work, we intend to improve our solution for activity recognition by including more activities (e.g., sitting/rising from a chair). We also will explore whether the performance and/or prediction time of the predictive model can be improved by selecting a subset of the kinematic measures, tuning the model’s parameters, and/or using a sliding window instead of taking into account each frame individually.

Considering our previous studies on Kinect-based gait analysis with Parkinson’s disease patients (based on manually identified gait cycles), we intend to evaluate the performance of the novel automatic gait analysis solution presented in the current contribution for gait-impaired patients.

## Supporting information

S1 Video Activity recognition and heel strike detection results. The video shows the results of gait analysis, namely of activity recognition and heel strike detection, for a trial of tasks T1, T2 and T3 performed by a given subject. From time 00:21 to 00:33 of the video, Kinect data acquired while the subject performed task T1 is shown in our *KiMA* application. The option for performing gait analysis is then selected, and the resulting detected activities and heel strikes are shown in *KiMA* as “Events” and “Labels” from 00:43 to 01:03. The same results are shown again with the video in slow motion from 01:03 to 02:13. Gait analysis is also performed for tasks T2 and T3, and the corresponding results are shown from 02:26 to 02:58, and 03:12 to 04:27, respectively.
(MP4)
